# Adaptive Marginal Median Filter for Colour Images

**DOI:** 10.3390/s110303205

**Published:** 2011-03-15

**Authors:** Samuel Morillas, Valentín Gregori, Almanzor Sapena

**Affiliations:** Instituto Universitario de Matemática Puray Aplicada, Universidad Politécnica de Valencia, Camino de Vera s/n, 46022 Valencia, Spain; E-Mails: vgregori@mat.upv.es (V.G.); alsapie@mat.upv.es (A.S.)

**Keywords:** robust filter, color image filter, vector median filter

## Abstract

This paper describes a new filter for impulse noise reduction in colour images which is aimed at improving the noise reduction capability of the classical vector median filter. The filter is inspired by the application of a vector marginal median filtering process over a selected group of pixels in each filtering window. This selection, which is based on the vector median, along with the application of the marginal median operation constitutes an adaptive process that leads to a more robust filter design. Also, the proposed method is able to process colour images without introducing colour artifacts. Experimental results show that the images filtered with the proposed method contain less noisy pixels than those obtained through the vector median filter.

## 1. Introduction

Noise is often introduced into digital images during the acquisition and transmission processes because of different reasons such as CCD sensor malfunction, transmission errors, storage faults, and difficult acquisition conditions. The presence of noise hampers the automatic processing of digital images and also affects their visualization quality. This implies that the noise reduction task, also known as image filtering, is a fundamental step in any computer vision system. In this context, several types of noise have been studied. Here we focus on the impulse noise case, which affects a portion of the image pixels, replacing their original values with other very different ones.

The earliest filters were developed to process gray-scale images and were based on linear approaches. However, it was found that nonlinear methods exhibit better performance and, in particular, that the median operator is the most robust method when the images are contaminated with impulse noise [1].

Recently, the interest in employing colour images has grown in a wide range of applications, which has motivated the development of colour image filters. The simplest attempts for processing colour images are based on applying a method for gray-scale images in each of the three colour channels independently [2]. However, it is well known that this way of processing is not appropriate for colour images because there exists a high correlation among the colour image channels which is not considered by this kind of methods [2,3]. For instance, the *Vector Marginal Median Filter* (VMMF) [2] uses the scalar median operator in each of the colour channels independently to obtain the filtered image. However, when obtaining the colour for a particular image pixel, the VMMF may combine components of image pixels that may be very different, which in turn may generate artificial colours (also known as artifacts). This implies that the VMMF is not useful for real applications since it cannot adapt its performance to the existence of correlation among the image channels.

From another point of view, the *Vector Median Filter* (VMF) [4] proposes to process the colour images by treating them as a vector field in order to take into account the interchannel correlation. The family of vector filters inspired by the VMF, which includes the *Directional Vector Filter* among others [3,5], is based on the theory of robust statistics [1,6]. The filters of this family, and specially the VMF, can perform quite robustly in impulse noise reduction without introducing colour artifacts, since they appropriately consider the colour components correlation. These filters select the vector that is closest to the rest of the vectors in a given population based on distance measure to be the output. However, the noise reduction capability of these filters is lower than the VMMF. For instance, in the case of a vector population where all vectors have one noisy component, the VMF will always select a noisy vector as output, since it cannot adapt to this extremely noisy situation.

In this work we propose a method to improve the performance of the VMF in this sense. Our method is based on applying a VMMF process over a group of pixels selected using the *Vector Median* (VM) of the population. As a result, a more robust adaptive filter design able to process colour images without introducing colour artifacts is obtained. As we will show in the experimental section, the images obtained through the proposed method contain less noisy pixels than those obtained through the VMF. The proposed method is intended to be used within more complex filtering procedures, for instance, in the noise reduction step, where the VMF is frequently used [7–9].

The paper is organized as follows. Section 2 recalls the basics of the VMMF and the VMF. The proposed filtering process is introduced in Section 3. Experimental results and comparisons are provided in Section 4 and, finally, Section 5 presents the conclusions.

## 2. Vector Marginal Median Filter and Vector Median Filter

Denote by **F** a colour (or multichannel) image to be processed and let *W* be a filtering window centered on the pixel under processing of size *N* × *N*, *N* = 3, 5, 7 … containing *N*^2^ = *n* pixels. The colour vectors in *W* are denoted as $F_{j}=(F_{j}^{R},\;F_{j}^{G},\;F_{j}^{B})$, *j* = 0, 1, …, *n* − 1, as usual in the RGB colour space. The distance between two vectors **F***_i_*, **F***_j_* is denoted as *ρ*(**F***_i_*, **F***_j_*). In this work, we take the Euclidean distance as the *ρ* function, but any other distance metric could be used instead [2,3,10–13].

### 2.1. Vector Marginal Median Filter

The output of the VMMF when processing the center pixel of *W* is the vector,

(1) $$\mathrm{VMM}=\left(\mathrm{med}\left(\left\{F_{0}^{R},\ldots ,\;F_{n-1}^{R}\right\}\right),\;\mathrm{med}\left(\left\{F_{0}^{G},\ldots ,\;F_{n-1}^{G}\right\}\right),\;\mathrm{med}\left(\left\{F_{0}^{B},\ldots ,\;F_{n-1}^{B}\right\}\right)\right)$$

where *med* denotes the statistical median operation. In this way, no relation among the colour components of **VMM** is considered, which leads to the problems mentioned in the previous section. On the other hand, **VMM** contains the most robust estimation in each component, which implies that its noise reduction capability is the highest.

### 2.2. Vector Median Filter

The VMF approaches the problem of noise reduction by looking for the most robust vector in the population. For this, each vector in the filtering window is associated with an accumulated distance to all other vectors which is computed as $R_{i}=\sum_{j=0}^{n-1}\rho (F_{i},\;F_{j})$. Thus, *R_i_* is the distance associated to the vector **F***_i_*. Then, the colour vectors are ordered according to *R_i_*, so that the ordering of the *R_i_*’s: *R*_(0)_ ≤ *R*_(1)_ ≤ … ≤ *R*_(*n*−1)_, implies the same ordering of the vectors **F***_i_*’s: **F**_(0)_ ≤ **F**_(1)_ ≤ … ≤ **F**_(*n*−1)_. Given this order, the output **VM** = **F**_(0)_, which is the colour vector associated to the minimum accumulated distance. Notice that because of the vector approach, the correlation among the **VM** components is considered, which avoids the generation of colour artifacts. However, in a very noisy context where all colour vectors contain some noisy component, **VM** will be noisy. In the following section we introduce a method intended to increase the noise reduction capability of the VMF in this sense.

## 3. Proposed Method: Adaptive Vector Marginal Median Filter

As mentioned above, the proposed method is based on the application of a VMMF operation over a selected group of colour vectors. To make this selection, we employ the **VM** of the filtering window *W* as follows.

Following the notation in Section 2, we take the vector median **VM** and we order the vectors in *W* according to their similarity with **VM**. So, we define the ordered set:

(2) $$W^{\prime }=\left\{F_{\left[0\right]},\;F_{\left[1\right]},\ldots ,\;F_{\left[n-1\right]}\right\}$$

such that

(3) $$\rho (\mathrm{VM},\;F_{\left[0\right]})\leq \rho (\mathrm{VM},\;F_{\left[1\right]})\leq \ldots \leq \rho (\mathrm{VM},\;F_{\left[n-1\right]})$$

where, obviously, **F**_[0]_ = **VM**.

We select a set of colour vectors, say *S*, which will be constituted by the *m* colour vectors most similar to **VM**. The objective of this selection is to adapt the performance of the method to the existence of correlation among the colour image channels. Notice that *m* is an adaptive parameter that relates **VM** with the number of pixels in *W* which are similar to it, which in turn are the colour vectors with a similar relation among their components. In this way,

(4) $$S=\left\{F_{\left[0\right]},\;F_{\left[1\right]},\ldots ,\;F_{\left[m-1\right]}\right\}$$

where *m* ≤ *n*. If *m* is low enough *m << n*, *S* contains the **VM** and *m* − 1 colour vectors with similar components. Therefore, *S* contains a set of robust colour vectors, for they are similar to the **VM** and, moreover, all the vectors in *S* have similar components. In an optimal situation, *m* should be set to the number of colour vectors in *W* similar to **VM**. However, in a very noisy context, some noisy components will be also included in *S*. Now, we apply the VMMF operation over the colour vectors in *S* to achieve high noise reduction so that the method can perform well in very noisy situations. The proposed method output is obtained as follows:

(5) $$\mathrm{AMM}=\left(\mathrm{med}\left(\left\{F_{\left[0\right]}^{R},\ldots ,\;F_{\left[m-1\right]}^{R}\right\}\right),\;\mathrm{med}\left(\left\{F_{\left[0\right]}^{G},\ldots ,\;F_{\left[m-1\right]}^{G}\right\}\right),\;\mathrm{med}\left(\left\{F_{\left[0\right]}^{B},\ldots ,\;F_{\left[m-1\right]}^{B}\right\}\right)\right)$$

In this way, we obtain a more robust vector than the **VM** because of the robustness of the median operation. Notice that now, given that the vectors in *S* are similar among them, which in turn implies that they have similar components, no colour artifacts will be generated. This happens because through the colour vector selection process the marginal median operation has been adapted to the similarities observed in the population, which overcomes the main drawback of the VMMF.

## 4. Experimental Results and Assessment

**Figure 1.**
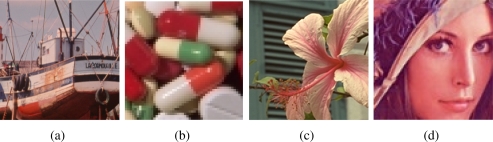
Test images: **(a)** Boats, **(b)** Pills, **(c)** Flower, and **(d)** Lenna.

Impulsive noise corruption process affects only some pixels in the image while leaving other pixels unchanged. Typically, the noise process changes one or more colour components of the affected pixel by replacing its original values with other values which usually significantly deviates from the originals. The kind of noise which is the most difficult to detect and remove assumes that the impulse is a random uniformly distributed value within the signal range. For RGB images, we consider that the noise is independently introduced in each of the three colour channels with probability *p*, which means that the (3*p* − 3*p*^2^ + *p*^3^) 100% image pixels are corrupted with noise. This noise model has been used to contaminate the test images in Figure 1.

The contaminated images have been filtered using the VMMF, VMF and the proposed AMMF. In the three cases we have used a 3 × 3 filtering window and we have filtered each image only once to better appreciate the performance differences. However, when the noise is high, several filtering processes are frequently necessary to obtain a totally clean image. Another possibility is to use a larger window size. For instance, it is known that using a 5 × 5 filtering window provides a higher noise reduction capability and could be more appropriate in general for filtering very noisy images. However, the method we propose is intended to be used within more complex filter designs in the noise reduction step, and most high performance advanced filters use a 3 × 3 filtering window [7–9]. Therefore, we consider that it is more interesting to study the performance of our method in this case.

The adaptive parameter *m* of AMMF should be set, in an optimal ideal situation, to the number of vectors similar to the vector median in each filtering window. Since we focus on extremely noisy situations, we expect this number to be quite low. Also, according to the filter design, as *m* increases, the performance of the proposed methods become more similar to the VMMF since more colour vectors are involved in the marginal median operation. This means that increasing *m* will increase both the noise reduction capability of the method and the likelihood to introduce colour artifacts in the output image (as we will see, numerical results in Tables 1–4 support this reasoning). Given that our objective is to improve the noise reduction capability of the VMF but avoiding as much as possible the introduction of colour artifacts, we have set *m* to the minimum value that makes sense to use: *m* = 3.

In addition to visual comparison, to assess the quality of the different filters we have used the objective quality measures *Mean Absolute Error* (MAE), *Peak Signal to Noise Ratio* (PSNR), and *Normalized colour Difference* (NCD) defined as follows [2,3]:

(6) $$\mathrm{MAE}=\frac{\sum_{i=1}^{N\cdot M}\sum_{q=1}^{Q}\left|F_{i}^{q}-{\hat{F}}_{i}^{q}\right|}{N\cdot M\cdot Q}$$

(7) $$\mathrm{PSNR}=20\;\text{log}\;\left(\frac{255}{\sqrt{\frac{1}{\mathrm{NMQ}}\sum_{i=1}^{N\cdot M}\sum_{q=1}^{Q}{\left(F_{i}^{q}-{\hat{F}}_{i}^{q}\right)}^{2}}}\right)$$

where *M*, *N* are the image dimensions, *Q* is the number of channels of the image (*Q* = 3 for colour images), and $F_{i}^{q}$ and ${\hat{F}}_{i}^{q}$ denote the *q*^th^ component of the original image vector and the filtered image, at pixel position *i*, respectively, and

(8) $${\mathrm{NCD}}_{\mathrm{Lab}}=\frac{\sum_{i=1}^{N\cdot M}{\Delta E}_{\mathrm{Lab}}}{\sum_{i=1}^{N\cdot M}E_{\mathrm{Lab}}^{*}}$$

where ${\Delta E}_{\mathrm{Lab}}={\left[{\left(\Delta L*\right)}^{2}+{\left(\Delta a*\right)}^{2}+{\left(\Delta b*\right)}^{2}\right]}^{\frac{1}{2}}$ denotes the perceptual colour error and $E_{\mathrm{Lab}}^{*}={\left[{\left(L*\right)}^{2}+{\left(a*\right)}^{2}+{\left(b*\right)}^{2}\right]}^{\frac{1}{2}}$ is the *norm* or *magnitude* of the original image colour vector in the L*a*b* colour space.

Also, to assess the robustness of the methods we have computed the percentage of noisy pixels (NP%) in the output images using the simple but effective method proposed in [14,15]. This detection method considers a colour pixel as noisy if it has less than *m* colour pixels within Euclidean distance lower than *d* in a 3 × 3 neighborhood. In particular we have set *m* = 2 and *d* = 35.

**Figure 2.**
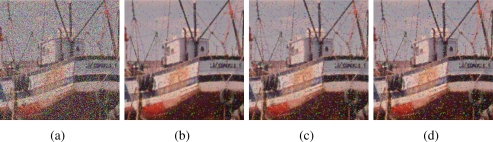
**(a)** Boats image corrupted with random-value impulse noise with *p* = 0.4 in each colour channel and outputs from: **(b)** VMMF, **(c)** VMF and **(d)** proposed AMMF.

**Figure 3.**
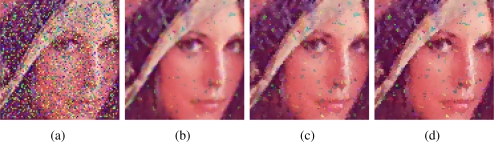
**(a)** Lenna image corrupted with random-value impulse noise with *p* = 0.3 in each colour channel and outputs from: **(b)** VMMF, **(c)** VMF and **(d)** proposed AMMF.

**Figure 4.**
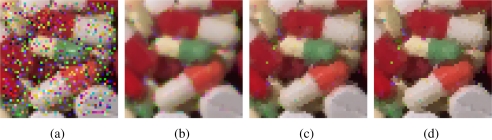
**(a)** Pills image corrupted with random-value impulse noise with *p* = 0.2 in each colour channel and outputs from: **(b)** VMMF, **(c)** VMF and **(d)** proposed AMMF.

**Figure 5.**
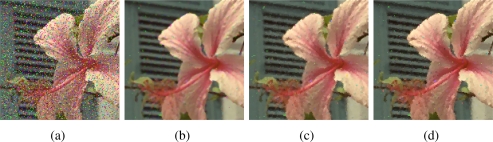
**(a)** Flower image corrupted with random-value impulse noise with *p* = 0.2 in each colour channel and outputs from: **(b)** VMMF, **(c)** VMF and **(d)** proposed AMMF.

Figures 2–5 show some noisy images and the respective filtering results using the VMMF, VMF and AMMF (*m* = 3). We can see that, overall, the highest noise reduction ability is exhibited by the VMMF. However, this filter also introduces many colour artifacts near edges (see for instance dark zones edges in Figure 2(b)). On the other hand, the images filtered with the AMMF contain less noisy pixels than those filtered with the VMF and no colour artifacts are introduced by any of these two methods. This fact is also illustrated in Tables 1–4 where we can see the NP for different images after filtering with the VMMF, VMF and AMMF*_m_* varying the value of *m* in {3, 4, 5}. In these tables AMMF obtains NP values lower than VMF. Also, we can see that when the noise is low (up to *p* = 0.20, which implies about 40% of noisy pixels) the images filtered with VMMF have higher NP than the AMMF and VMF. This happens because although VMMF has the higher ability to suppress noise it also generates colour artifacts, which increases NP in the output images. This fact implies that VMMF is not reliable and should not be used in practical applications. When the noise is high (*p* ≥ 0.30, which implies more than 50% of noisy pixels), VMMF still introduces artifacts but NP is lower because the percentage of noise reduced is much larger than the artifacts introduced. With respect to the rest of the quality measures, *i.e.*, MAE, PSNR and NCD, the best results are obtained by VMMF, but notice that these measures do not consider specifically the introduction of colour artifacts. When the noise is high, AMMF outperforms VMF in terms of MAE and NCD. From the visual results and the NP, it would be logical that AMMF always outperforms VMF, but these measures do not perfectly match with these criteria in such a noisy context. Finally, if we analyze the performance of AMMF for the different values of *m* considered, we can see that the best numerical results are obtained for *m* = 4. However, increasing the value of *m* also increases the likelihood to introduce colour artifacts, so we prefer to set *m* = 3. Most probably, *m* = 4 achieves the best trade-off between noise suppression and artifact generation, which implies better numerical values, but since it is a primary objective to avoid the generation of artifacts, we set *m* = 3. These results allow us to conclude that AMMF (*m* = 3) is the method able to obtain the most robust and reliable results since its ability to suppress noise is higher than VMF and it does not generate colour artifacts, which is an advantage with respect to VMMF.

## 5. Conclusions

In this paper we have presented an adaptive method for impulse noise reduction in colour images whose objective is to improve the noise reduction capability of the classical vector median filter. The filter is based on the selection of a few vectors in a population using the vector median and the application of a vector marginal median filtering over the selected vectors. The robustness and reliability of the method is achieved because the selection of vectors adapts the performance of the marginal median operation to an appropriate context. Experimental results show that the images filtered with the proposed method contain less noisy pixels than those obtained through the vector median filter. Also, the proposed method is able to process colour images without introducing colour artifacts, which is an advantage with respect to the vector marginal median filter. These results suggest that a more robust correlated filtering method might be obtained, which opens an interesting line of research. The proposed method can be used within more complex filtering procedures, for instance, in the noise reduction step, where the VMF is frequently used.

## Figures and Tables

**Table 1.** Performance comparison in terms of MAE, PSNR, and NCD when filtering the Boats image contaminated with random-value impulse noise with probability *p* in each colour channel.

Filter	*p* = 0.10				*p* = 0.20				*p* = 0.30				*p* = 0.40			
	MAE	PSNR	NCD	**NP**%	MAE	PSNR	NCD	**NP**%	MAE	PSNR	NCD	**NP**%	MAE	PSNR	NCD	**NP**%

None	7.55	18.82	12.02	22.2	15.01	15.83	22.60	40.5	22.70	14.03	32.28	57.9	30.26	12.77	40.40	73.2
VMMF	4.30	29.99	3.74	0.9	5.24	28.07	5.06	2.1	6.78	25.55	7.31	3.7	9.52	22.56	11.13	6.5
VMF	4.67	29.51	3.19	0.7	6.18	26.79	4.72	2.0	8.82	23.44	8.29	5.2	12.93	20.32	13.76	10.2
AMMF_3_	4.73	29.09	3.28	0.7	6.01	26.53	4.58	1.8	8.18	23.24	7.75	4.8	11.86	20.08	13.13	9.8
AMMF_4_	4.89	29.13	3.33	0.6	5.97	26.88	4.55	1.5	7.99	23.68	7.60	3.9	11.59	20.47	12.86	8.3
AMMF_5_	4.77	29.02	3.41	0.9	5.68	27.03	4.63	1.9	7.43	23.98	7.37	4.6	10.73	20.79	12.30	9.1

**Table 2.** Performance comparison in terms of MAE, PSNR, and NCD when filtering the Lenna image contaminated with random-value impulse noise with probability *p* in each colour channel.

Filter	*p* = 0.10				*p* = 0.20				*p* = 0.30				*p* = 0.40			
	MAE	PSNR	NCD	**NP**%	MAE	PSNR	NCD	**NP**%	MAE	PSNR	NCD	**NP**%	MAE	PSNR	NCD	**NP**%

None	7.63	18.52	11.33	22.4	15.19	15.56	21.37	41.4	22.94	13.75	30.20	57.2	30.31	12.61	37.79	71.9
VMMF	4.85	28.58	4.17	1.5	6.06	26.72	5.84	2.6	7.97	24.26	8.38	4.1	10.70	21.86	11.67	6.4
VMF	5.25	28.05	3.45	1.2	7.15	25.60	5.18	2.6	10.05	22.50	8.68	6.3	14.20	19.82	13.54	10.3
AMMF_3_	5.44	27.57	3.66	1.0	7.04	25.36	5.16	2.4	9.36	22.32	8.23	5.7	12.98	19.64	12.83	10.2
AMMF_4_	5.63	27.64	3.73	0.9	7.00	25.78	5.09	1.9	9.04	22.78	8.05	5.0	12.58	20.00	12.49	9.0
AMMF_5_	5.39	27.69	3.75	1.2	6.55	26.00	5.12	2.2	8.38	23.10	7.84	5.4	11.77	20.27	12.08	9.5

**Table 3.** Performance comparison in terms of MAE, PSNR, and NCD when filtering the Pills image contaminated with random-value impulse noise with probability *p* in each colour channel.

Filter	*p* = 0.10				*p* = 0.20				*p* = 0.30				*p* = 0.40			
	MAE	PSNR	NCD	**NP**%	MAE	PSNR	NCD	**NP**%	MAE	PSNR	NCD	**NP**%	MAE	PSNR	NCD	**NP**%

None	7.46	18.66	11.89	25.1	14.72	15.64	22.71	40.4	21.94	13.85	30.92	55.7	29.56	12.56	38.96	70.0
VMMF	5.35	26.98	4.91	5.0	7.01	25.30	7.23	7.2	9.71	22.65	10.01	8.3	13.04	20.77	14.26	12.0
VMF	5.97	26.55	3.84	3.4	8.21	24.30	5.93	5.1	12.17	21.17	10.10	7.4	16.24	19.29	15.62	13.0
AMMF_3_	6.27	25.95	4.19	2.7	8.30	23.70	6.24	4.6	11.47	20.92	9.82	7.3	14.95	19.17	15.10	12.6
AMMF_4_	6.66	25.89	4.36	2.3	8.41	23.93	6.23	4.2	11.26	21.25	9.42	5.9	14.49	19.54	14.53	12.0
AMMF_5_	6.47	25.75	4.52	3.2	7.97	24.03	6.40	4.5	10.58	21.43	9.56	7.6	13.48	19.87	14.09	12.6

**Table 4.** Performance comparison in terms of MAE, PSNR, and NCD when filtering the Flower image contaminated with random-value impulse noise with probability *p* in each colour channel.

Filter	*p* = 0.10				*p* = 0.20				*p* = 0.30				*p* = 0.40			
	MAE	PSNR	NCD	**NP**%	MAE	PSNR	NCD	**NP**%	MAE	PSNR	NCD	**NP**%	MAE	PSNR	NCD	**NP**%

None	7.46	18.82	11.57	22.8	14.70	15.88	21.63	41.4	22.33	14.08	30.87	58.8	29.70	12.86	38.77	73.5
VMMF	5.00	28.63	3.89	1.2	6.25	26.81	5.72	2.0	8.06	24.56	7.90	3.5	10.87	22.05	11.00	5.9
VMF	5.42	28.17	2.97	1.0	7.46	25.63	4.54	1.9	10.19	22.84	7.71	5.4	14.20	20.14	12.49	10.0
AMMF_3_	5.56	27.76	3.14	1.0	7.38	25.38	4.65	1.9	9.53	22.73	7.61	5.0	13.10	19.98	12.51	9.5
AMMF_4_	5.87	27.85	3.32	0.8	7.32	25.77	4.70	1.4	9.26	23.20	7.58	4.1	12.72	20.33	12.34	7.9
AMMF_5_	5.73	27.77	3.44	1.1	6.86	25.95	4.88	1.9	8.62	23.47	7.65	4.6	11.86	20.64	12.07	8.8
